# Associations Between the Use of eHealth and Out-of-Hours Services in People With Type 1 Diabetes: Cross-Sectional Study

**DOI:** 10.2196/13465

**Published:** 2019-03-21

**Authors:** Anne Helen Hansen, Tor Claudi, Eirik Årsand

**Affiliations:** 1 Centre for Quality Improvement and Development University Hospital of North Norway Tromsø Norway; 2 Department of Community Medicine Faculty of Health Sciences UiT The Arctic University of Norway Tromsø Norway; 3 Department of Medicine Nordland Hospital Bodø Norway; 4 Norwegian Centre for E-health Research University Hospital of North Norway Tromsø Norway; 5 Department of Clinical Medicine UiT The Arctic University of Norway Tromsø Norway

**Keywords:** eHealth, internet, health care utilization, out-of-hours services, cross-sectional study, diabetes mellitus, type 1, Norway

## Abstract

**Background:**

Despite the increasing prevalence of diabetes and the increasing use of eHealth, little is known about the association between provider-based health services and eHealth among people with diabetes. This is the second study in a project exploring the associations between the use of eHealth and the use of provider-based health services.

**Objective:**

The objective of this study was to investigate which eHealth services are used among out-of-hours (OOH) visitors with type 1 diabetes (T1D), and whether the use of eHealth (eg, apps, search engines, video services, and social media) was associated with the use of OOH services. We also wanted to investigate associations between anxiety, reassurance, and change in doctor-seeking behavior because of health information acquired from the Internet, and the use of OOH services.

**Methods:**

We used data from a 2018 email survey of members of the Norwegian Diabetes Association (18-89 years old). Respondents with T1D were eligible for analyses. Using descriptive statistics, we estimated the use of OOH services and eHealth. Using logistic regressions, we studied the associations between the use of OOH services and the use of eHealth, as well as associations between the use of OOH services and reported consequences of using Internet-based health information.

**Results:**

In the sample of 523 people with T1D (mean age 47 years), 26.7% (129/484) visited OOH services once or more during the previous year. Among the OOH visitors, search engines were used for health purposes by 86.7% (111/128), apps (health apps in general) by 63.6% (82/129), social media by 45.3% (58/128), and video services by 28.4% (36/127). The use of OOH services was positively associated with self-reported anxiety/depression (odds ratio [OR] 4.53, 95% CI 1.43-14.32) and with the use of apps (OR 1.73, 95% CI 1.05-2.85), but not with other types of eHealth. Those who had felt anxious based on information from the Internet were more likely to visit OOH services compared with those who had not felt anxious (OR 2.38, 95% CI 1.50-3.78). People who had decided to consult a doctor based on information from the Internet were more likely to visit OOH services (OR 2.76, 95% CI 1.64-4.66), compared to those who had not made such an Internet-based decision.

**Conclusions:**

People with T1D were frequent users of OOH services, and the OOH visitors were frequent users of eHealth. The use of OOH services was positively associated with the use of health apps, with self-reported anxiety/depression, and with feeling anxious based on information from the Internet. Likewise, deciding to consult a doctor based on information from the Internet was positively associated with OOH visits. The use of eHealth seems to have a significant impact on people with T1D.

## Introduction

### Increasing Prevalence of Diabetes

The prevalence of diabetes is increasing worldwide. Global prevalence in adults is estimated at 8.8% [1] and the Norwegian prevalence at 4.7% [2]. Around 245,000 people are diagnosed with diabetes in Norway. Of these, around 28,000 have type 1 diabetes (T1D) [2]. Patients represent a large proportion of health care contacts, and diabetes is a considerable burden on patients regarding morbidity and mortality [3]. Most patients do not reach the combined national treatment targets for prevention of complications [4-6].

### Consequences of Increasing Use of eHealth Services

Electronic health (eHealth) is defined as “the transfer of health resources and health care by electronic means” [7]. Most Norwegian households (98%) had Internet access and access to a smart phone in 2017 [8,9]. Use of the Internet for health purposes has increased rapidly in the past decades [10-12], and 75%-80% of Internet users in the United States and Europe report conducting health-related searches [10-13]. Our first study in this project found that 87% of Norwegians with T1D used eHealth in one or more forms, exceeding the use of about 78% in the general population [14,15]. Although T1D is a prevalent chronic and challenging disease, the consequences of patients seeking health information from the Internet have not been comprehensively explored. Medlock et al studied the consequences of health information seeking among seniors in the Netherlands, finding that 38% had felt anxiety and 56% had felt reassured. Some had changed their plans regarding doctor visits: 48% had decided to go to the doctor, and 24% had decided *not* to go based on health information from the Internet [16].

### Norwegian Health Care and Use of Out-of-Hours Services

Norwegian health care is based on universal insurance. However, consultations for adults are co-paid through a small fee [14]. Primary health care, including a regular general practitioner (GP) and GP-based out-of-hours (OOH) services, is provided to all residents by the municipalities through the patient list system. This includes almost all Norwegian residents [17]. Specialist services are operated by regional and local health enterprises owned by the national government. Access to specialist care is usually through referral from GPs or OOH services (the gatekeeper role). People with T1D are recommended to visit specialist services at least annually. There are indications that many patients miss this check-up [14]. We know little about the extent to which people with T1D visit Norwegian OOH services, but we know that people with any type of diabetes and people with chronic disease constitute a significant proportion of visitors [18]. Regarding the general population, 27.4% of listed patients contacted OOH services in 2008 (all contact types) [19], and 17% of the population visited OOH services in 2017 [20]. Norway has a high OOH contact rate compared to other countries, and 75% of contacts have been classified as non-urgent [21].

### Associations Between Use of eHealth and Use of Out-of-Hours Services

Research on the association between the use of eHealth and provider-based health care is scarce [22,23]. However, frequent users of health services seem more likely to seek health information on the Internet compared with non-users [12]. This study is a part of the DIAcare project, aiming to investigate associations between eHealth and different provider-based health care services. The project also aims to investigate the associations between socioeconomic status and the use of eHealth, whether eHealth information is discussed in the clinical encounter, and whether the use of eHealth might lead to (or prevent) doctor visits [24]. Our first study in this project revealed no association between the use of eHealth and GP visits for T1D patients, whereas we found a positive association between the use of search engines and somatic specialist services [14]. This second study focuses on the association between the use of eHealth and the use of OOH services in persons with T1D. Understanding the associations between eHealth and the use of provider-based health services is important for patients as well as for health care providers, policy makers, and society, in order to enable evidence-based planning for future health care services in a society where eHealth is increasingly used.

### Aim

The aim of this study was to investigate which eHealth services were used among OOH visitors with T1D, and the associations between the use of eHealth (ie, health apps in general, search engines, video services, and social media) and the use of OOH services. Furthermore, we aimed to investigate whether anxiety, reassurance, and change of doctor-seeking behavior because of the use of eHealth were associated with the use of OOH services.

## Methods

### Data

This cross-sectional study used data obtained in 2018 from members of the Norwegian Diabetes Association (NDA). As of December 31, 2017, the organization had 33,908 members, of which about 30% have T1D [25]. The Norwegian Social Science Data Service (NSD) Web survey distributed the invitations to a randomly selected sample of 5971 individuals who had their email addresses recorded by NDA. Initially, we planned to use data from the seventh Tromsø Study, conducted in 2015/2016 [24]. However, the Tromsø Study could not give us access, and we developed a tailored questionnaire based on the specific objectives of our study, using relevant questions from other published surveys [26,27].

Information about the study was posted together with the invitation. The questionnaire (Multimedia Appendix 1) included questions about health status including specific questions about disease duration, severity and treatment of diabetes, use of and experiences with eHealth and health care services, as well as demographic and socioeconomic information. We reviewed and tested the questionnaire several times before distribution to the informants. Non-respondents were given one reminder, submitted by email 15 days after the first request.

### Participants

The respondents could not fill in the questionnaire more than once. Starting from 1250 participants, we first excluded the 66 individuals who did not suffer from diabetes themselves (eg, family members, health personnel, and others). We also excluded those who failed to respond to most of the questions (n=5), those who did not give information about gender (n=93), and participants with diabetes types other than T1D (n=563). The analyzed sample consisted of 523 respondents with T1D. For a flowchart of the study population, we refer to our previous study [14].

### Variables

The dependent variable in all analyses was the use of OOH services once or more during the previous 12 months. The use of eHealth was subdivided into 4 variables: apps for mobile phone or tablet computers (health apps in general, not necessarily diabetes self-care apps), search engines (eg, Google), social media (eg, Facebook), and video services (eg, YouTube). We dichotomized these variables by merging the original four response options into “never or once” and “sometimes or often,” in line with previous research [16].

The following questions were also asked: “Based on information from the Internet, have you felt anxious/felt reassured/decided to consult a doctor when you would otherwise *not* have consulted one/decided *not* to consult a doctor when you would otherwise have consulted one?” The answering options “no, once, sometimes, or often” were merged into “no” and “once, sometimes or often” for an easier interpretation of logistic regressions. Since we were interested in whether changes in decisions had ever taken place, we placed “no” in a separate category.

Age was grouped in 20-year age groups. The education categories were labeled low (primary/part of secondary school), middle (high school), high (college/university <4 years), and highest (college/university 4 years or more). Response options for self-rated health were excellent, good, fair, bad, and very bad. We merged the bad and very bad categories due to low numbers in the very bad category (4 respondents). Response options for self-reported degree of anxiety/depression were none, slight, moderate, severe, and extreme. We merged the severe and extreme categories due to only 1 respondent in the extreme category.

Since the use of health apps in general turned out to be an issue in this study, we wanted to inform the readers about the answers obtained from the following question in the questionnaire: “During the past 12 months, have you used apps for smartphone or tablet computer for follow-up of your own diabetes?” The answering options were “never, less than once a month, once a month, once a week, and every day.” According to the study protocol, this variable was not included in the regression analyses.

Due to the relatively low participation rate, we constructed a dichotomous response time variable in order to compare late respondents with early respondents, assuming that the late respondents were more similar to non-respondents [28]. All participants who responded initially were placed in one group, and those who responded after the reminder were placed in the other group.

### Analyses

Data were analyzed by means of descriptive statistics and logistic regressions. Correlations were tested with Spearman correlation test.

Use of OOH services was the dependent variable in all the analyses. In the first multivariate regression, the independent variables were the use of apps (general health apps), search engines, social media, and video services, gender, age, education, self-rated health, and self-reported degree of anxiety/depression. For the second set of analyses, we performed four univariate and four multivariate regression analyses. The independent variables in the four univariate analyses were “felt anxious,” “felt reassured,” “decided to consult a doctor when you would otherwise *not* have consulted one,” and “decided *not* to consult a doctor when you would otherwise have consulted one.” These were key independent variables in the multivariate analyses, which we adjusted for gender, age, education, and self-rated health. All the independent variables were introduced collectively into the multivariate models.

We compared those who did not respond to our initial inquiry, but eventually consented, with the early respondents. This was done by subsequently introducing the response time variable into the regression models. We used 95% confidence intervals (CI) throughout the study. All analyses were accomplished using Stata, version 14.2.

### Ethics

The Regional Committee for Medical and Health Research Ethics (REK) found that an application for this project was not required according to the Norwegian Health Research Act (ref 2015/1779/REK nord). The data protection officer (Personvernombudet) at the University Hospital of North-Norway approved the study (ref. 2017/6579). The NSD data bureau received no information about the participants other than the email addresses.

## Results

### Participation

In total, 1250 persons aged 18-89 years answered the questionnaire, constituting a minimum response rate of 20.9%. We assume the real response rate to be higher since we had more than 400 bounce backs from email servers unable to deliver the invitation, and we do not know how many actually received the survey email. Eligible for analysis were the 523 persons who reported to have T1D.

### Characteristics of Users of Out-of-Hours Services

Among the users of OOH services, the largest groups were men (66/129, 51.2%), people aged 40-59 years (53/129, 41.1%), married/cohabitants (87/99, 87.9%), people employed full-time or part-time (81/129, 62.8%), people with the highest education (41/128, 32.0%), the highest household income (63/126, 50.0%), good self-rated health (59/128, 46.1%), good self-rated regulation of diabetes (66/127, 51.9%), no anxiety/depression (71/128, 55.5%), and who had lived with diabetes for 30 years or more (43/129, 33.3%) (Table 1).

Based on information from the Internet, 44.5% (57/128) of OOH users reported that they had felt anxious, 55.9% (71/127) had felt reassured, 28.7% (37/129) had decided to consult a doctor when they would otherwise not have consulted one, whereas 21.9% (28/128) had decided *not* to consult a doctor when they would otherwise have consulted one (Table 1).

Mean age among the users of OOH services was 47.2 years, 44.3 years for women, and 50.0 years for men. Median age was 48 years. Mean disease duration was 21.9 years (median 20 years).

### Use of Out-of-Hours Services and eHealth

During the previous year, 26.7% (129/484) visited OOH services once or more. Men visited slightly more than women (29.1% versus 24.5%), and people aged 60 years and over visited slightly more than younger age groups (Table 2).

Among the OOH users, 63.0% (80/127) visited once and 26.8% (34/127) visited twice during the previous year. Only 2.4% (3/127) visited 5 times or more.

In the total sample as well as among the OOH users, search engines were the most widely used form of eHealth, followed by the use of health apps (all kinds). Among the OOH users, search engines were used by 86.7% (111/128), health apps (all kinds) by 63.6% (82/129), social media by 45.3% (58/128), and video services by 28.4% (36/127). Users of OOH services used apps slightly more than the total sample (Table 3).

Among the OOH users who reported having used health apps (all kinds) sometimes or often, the largest group (32/82, 39%) had never used apps for self-care of their diabetes, and the second largest group (28/82, 34%) had used apps for self-care less than once a month. Only 11% (9/82) had used apps for diabetes self-care every day (Table 4).

### Associations Between Use of Out-of-Hours Services and eHealth

Visits to OOH services were positively associated with the use of health apps (odds ratio [OR] 1.73, 95% CI 1.05-2.85), but not associated with the use of any other type of eHealth. OOH services visits were positively associated with self-reported moderate or severe anxiety/depression, compared with no anxiety/depression (OR 2.18, CI 1.04-4.54, and OR 4.53, CI 1.43-14.32, respectively). Gender, age, education, and self-rated health were not associated with the use of OOH services (Table 5).

**Table 1:** Characteristics of total T1D sample and sample using out-of-hours services once or more during the previous 12 months.

Characteristics		Total sample % (n/N)	Users of OOH services % (n/N)
**Gender**			
	Female	53.7 (281/523)	48.8 (63/129)
	Male	46.3 (242/523)	51.2 (66/129)
**Age**			
	18-39 years	34.0 (178/523)	32.5 (42/129)
	40-59 years	42.6 (223/523)	41.1 (53/129)
	60+ years	23.4 (122/523)	26.4 (34/129)
**Marital status**			
	Single	11.0 (42/380)	12.1 (12/9)
	Married/cohabitant	89.0 (338/380)	87.9 (87/99)
**Main daily activity**			
	Working^a^	64.0 (308/481)	62.8 (81/129)
	Pensioner, old age	13.5 (65/481)	14.0 (18/129)
	Pensioner, disability	11.0 (53/481)	10.1 (13/129)
	Pupil/student	7.3 (35/481)	7.0 (9/129)
	Other	4.2 (20/481)	6.1 (8/129)
**Education^b^**			
	Low	8.1 (39/480)	11.0 (14/128)
	Middle	29.0 (139/480)	28.9 (37/128)
	High	31.7 (152/480)	28.1 (36/128)
	Highest	31.2 (150/480)	32.0 (41/128)
**Household income^c^**			
	Low	14.1 (66/467)	16.7 (21/126)
	Middle	34.9 (163/467)	33.3 (42/126)
	High	51.0 (238/467)	50.0 (63/126)
**Duration of diabetes**			
	<10 years	24.3 (127/522)	27.9 (36/129)
	10-19 years	20.5 (107/522)	20.2 (26/129)
	20-29 years	19.4 (101/522)	18.6 (24/129)
	30 years and over	35.8 (187/522)	33.3 (43/129)
**Self-rated regulation of diabetes**			
	Excellent	19.4 (101/520)	20.5 (26/127)
	Good	56.2 (292/520)	51.9 (66/127)
	Fair	19.8 (103/520)	20.5 (26/127)
	Bad/very bad	4.6 (24/520)	7.1 (9/127)
**Self-rated health**			
	Excellent	17.9 (93/521)	14.1 (18/128)
	Good	51.6 (269/521)	46.1 (59/128)
	Fair	21.7 (113/521)	28.9 (37/128)
	Bad/very bad	8.8 (46/521)	10.9 (14/128)
**Degree of anxiety/depression**			
	None	65.0 (334/514)	55.5 (71/128)
	Slight	22.2 (114/514)	21.9 (28/128)
	Moderate	9.5 (49/514)	16.4 (21/128)
	Severe	3.3 (17/514)	6.2 (8/128)
**Based on information from the Internet, have you:**			
	Felt anxious (yes, once/sometimes/often)	31.8 (155/487)	44.5 (57/128)
	Felt reassured (yes, once/sometimes/often)	54.6 (263/482)	55.9 (71/127)
	Decided to consult a doctor when you would otherwise not have consulted one (yes, once/sometimes/often)	17.5 (87/497)	28.7 (37/129)
	Decided not to consult a doctor when you would otherwise have consulted one (yes, once/sometimes/often)	18.3 (90/493)	21.9 (28/128)

^a^Full-time or part-time.

^b^Low (primary/part of secondary school), middle (high school), high (college/university <4 years), highest (college/university ≥4 years).

^c^Low (350,000 NOK or less), Middle (351,000-750,000 NOK), High (751,000 NOK or more).

**Table 2:** Proportion using out-of-hours services once or more during the previous 12 months, according to gender and age.

Characteristics		n/N	%	95% CI
Total sample		129/484	26.7	22.7-30.6
**By gender**				
	Female	63/257	24.5	19.2-29.8
	Male	66/227	29.1	23.5-35.3
**By age, years**				
	18-39	42/156	26.9	19.9-33.9
	40-59	53/211	25.1	19.2-31.0
	60+	34/117	29.1	20.8-37.3

**Table 3:** Proportion using different kinds of eHealth sometimes or often during the previous 12 months.

Variables	Total sample			Users of out-of-hours services		
	n/N	%	95% CI	n/N	%	95% CI
Health apps (all kinds)	285/514	55.5	51.1-59.7	82/129	63.6	54.8-71.5
Search engines	431/513	84.0	80.6-86.9	111/128	86.7	79.6-91.6
Social media	232/513	45.2	40.9-49.6	58/128	45.3	36.8-54.1
Video services	118/506	23.3	19.8-27.2	36/127	28.4	21.1-36.9

**Table 4:** Proportion using apps for self-care of diabetes during the previous 12 months.

Frequency	Total sample (N=509)			Users of out-of-hours services (N=127)			Users of out-of-hours services and apps (N=82)		
	n	%	95% CI	n	%	95% CI	n	%	95% CI
Never	282	55.4	51.0-59.7	62	48.8	40.1-57.6	32	39.0	28.9-50.2
<1x per month	135	26.5	22.9-30.5	36	28.4	21.1-36.9	28	34.1	24.5-45.2
1x per month	41	8.1	6.0-10.8	10	7.9	4.3-14.1	9	11.0	5.7-20.0
1x per week	21	4.1	2.7-6.3	7	5.5	2.6-11.2	4	4.9	1.8-12.5
Every day	30	5.9	4.1-8.3	12	9.4	5.4-16.0	9	11.0	5.7-20.0

**Table 5:** Probability of using out-of-hours services once or more during the previous 12 months in a population with diabetes type 1 (multivariate logistic regressions).

Variables		Use of out-of-hours services (yes/no)		
		OR	*P*	95% CI
**Health apps** **(all kinds)**				
	Never/once^a^	1.00		
	Sometimes/often	*1.73* ^b^	*.03* ^b^	*1.05-2.85* ^b^
**Search engines**				
	Never/once^a^	1.00		
	Sometimes/often	1.17	.67	0.57-2.40
**Social media**				
	Never/once^a^	1.00		
	Sometimes/often	0.76	.29	0.46-1.26
**Video services**				
	Never/once^a^	1.00		
	Sometimes/often	1.23	.45	0.72-2.10
**Gender**				
	Female^a^	1.00		
	Male	1.26	.31	0.81-1.96
**Age**				
	18-39^a^	1.00		
	40-59	0.88	.63	0.53-1.47
	60+	1.32	.36	0.73-2.36
**Education^b^**				
	Low^a^	1.00		
	Middle	0.71	.44	0.31-1.65
	High	0.65	.32	0.28-1.51
	Highest	0.82	.65	0.35-1.91
**Self-rated health**				
	Excellent^a^	1.00		
	Good	1.15	.66	0.62-2.15
	Fair	1.53	.25	0.75-3.15
	Bad/very bad	1.26	.63	0.50-3.16
**Self-reported anxiety/depression**				
	None	1.00		
	Slight	1.13	.65	0.66-1.96
	Moderate	2.18	.04	1.04-4.54
	Severe	*4.53* ^b^	*.01* ^b^	*1.43-14.32* ^b^

^a^Reference groups.

^b^Low (primary/part of secondary school), Middle (high school), High (college/university < 4 years), Highest (college/university 4 years or more.

**Table 6:** Probability of using out-of-hours services once or more during the previous 12 months according to reported effects of using the Internet for health information (univariate and multivariate logistic regressions).

Variables		Use of out-of-hours services (univariate logistic regressions)			Use of out-of-hours services (multivariate logistic regressions)^a^		
		OR	*P*	95% CI	OR	*P*	95% CI
**Based on information from the Internet, have you:**							
	Felt anxious^b^	*2.13* ^c^	*<.001* ^c^	*1.40-3.25* ^c^	*2.38* ^c^	*<.001* ^c^	*1.50-3.78* ^c^
	Felt reassured^b^	1.04	.83	0.70-1.58	1.21	.39	0.79-1.86
	Decided to consult a doctor, when you would otherwise not have consulted one^b^	*2.7* 0^c^	*<.001* ^c^	*1.65-4.42* ^c^	*2.76* ^c^	*<.001* ^c^	*1.64-4.66* ^c^
	Decided not to consult a doctor, when you would otherwise have consulted one^b^	1.34	.25	0.81-2.21	1.33	.29	0.79-2.27

Statistically significant findings are marked in italics.

^a^adjusted for gender, age, education, and self-rated health.

^b^0=no (reference), 1=once, sometimes, or often.

### Associations Between Use of Out-of-Hours Services and Reported Effects of Using the Internet for Health Information

Those who had felt anxious based on information from the Internet were more than twice as likely to visit OOH services compared with those who had not felt anxious, both in univariate (OR 2.13, CI 1.40-3.25) and in multivariate regression models (OR 2.38, CI 1.50-3.78). People who had decided to consult a doctor based on Internet information (when they would otherwise *not* have consulted one) were almost three times as likely to visit OOH services (OR 2.70, CI 1.65-4.42, and OR 2.76, CI 1.64-4.66, for univariate and multivariate regressions, respectively), compared with those who had not changed their decision to consult a doctor.

Feeling reassured based on information from the Internet was not associated with the use of OOH services. Likewise, we found no association between deciding *not* to consult a doctor (when they would otherwise have consulted one) and the use of OOH services (Table 6).

All findings presented in Tables 5 and 6 persisted after including the response time variable in the regression analyses. There were no strong correlations (defined as Spearman rho >.5) between the independent variables in any of the models.

## Discussion

### Principal Findings

We found that 26.7% (129/484) of people with T1D visited OOH services once or more during the previous year. Search engines were used sometimes or often by 86.7% (111/128) of the OOH visitors, apps by 63.6% (82/129), social media by 45.3% (58/128), and video services by 28.4% (36/127). Visits to OOH services were positively associated with self-reported anxiety/depression and with the use of health apps, but not with other forms of eHealth. Among those who had used OOH services as well as health apps during the previous year, 39% (32/82) had never used apps for self-care of their diabetes. Those who had felt anxious based on information from the Internet were more than twice as likely to visit OOH services compared with those who had not felt anxious. People who had decided to consult a doctor based on information from the Internet (when they would otherwise not have consulted one) were almost three times as likely to visit OOH services compared with those who had not made a decision to consult a doctor based on information from the Internet.

### Extensive Use of Out-of-Hours Services by People With T1D

Among people with T1D, 26.7% reported one or more visit to OOH services during the previous year. Sandvik et al reported 27.4 contacts with OOH services per 100 list patients in Norway (general population, 2008) [19]. Unlike our rate, this rate includes more than just visits (ie, house calls, telephone contacts, and simple contacts), indicating a higher visit rate among people with T1D compared with the general population. The National Centre for Emergency Primary Health Care reported 1,332,024 visits to OOH services in Norway in 2017 [29], indicating a rate of 25.3% in a population of 5,258,317 inhabitants (January 1, 2017). Children, adolescents, and tourists were included, as well as the number of visits for those who visited more than once, meaning that our rate solely for visits is higher. Statistics Norway found that 17.0% of the population (all ages, children included) consulted OOH services in 2017 [20]. Since children and adolescents are frequent OOH users [29], the adult general population rate is probably lower. This is in line with data collected by the Tromsø Study, suggesting a preliminary general population OOH visit rate of 13.5% (2740/20,294) among people aged 40 years and over [27]. Despite constraints regarding comparison with these figures, we conclude that our rate for OOH visits among people with T1D is higher than these general population rates. Our findings contribute to underpinning previous documentation that people with diabetes are frequent users of OOH services in Norway [18,29]. Research from other countries confirms that this is an international phenomenon. Diabetes was the most frequent chronic somatic disorder among frequent attenders in the Netherlands in 2007 [30], and patients with diabetes accounted for almost 1 out of 10 emergency department visits in the United States (2010) [31].

### Health Apps Widely Used by Attenders of Out-of-Hours Services

In the first paper in this project, we documented that people with T1D are frequent users of all the investigated four forms of eHealth [14]. OOH attenders with T1D used all forms of eHealth equally or more than the total T1D sample, and the largest difference was found for apps (Table 3). This extensive use is in line with the illness behavior model stating that people in poorer health more likely seek Web-based disease-related information [32]. It is also in line with a German study reporting that app users were more likely to report chronic conditions, such as diabetes [33].

### Low Daily Use of Diabetes Self-Care Apps

Most of the combined OOH and app users had used apps for self-care of their diabetes; however, only 11.0% of them used diabetes self-care apps every day (Table 4). Only 5.9% of the total T1D sample used diabetes self-care apps every day, which is a low percentage compared to 24% in adults with T1D reported in a recent Australian study [34]. They found that the use of self-care apps was positively associated with female gender and lower age. More female participants and a lower mean age in the Australian study may explain some of the differences from our rate. However, the study methodology and question wording vary, and the figures are not directly comparable.

### Positive Associations Between Out-of-Hours Services Visits and Use of Health Apps and Self-Reported Anxiety or Depression

The possibility of visiting OOH services was higher for persons with T1D who used health apps (all kinds), compared with those who did not (Table 5). We found no other studies investigating this relation.

Moderate and severe self-reported anxiety/depression was more prevalent among the users of OOH services, compared with the total sample (Table 1). Severe anxiety/depression increased the possibility of visiting OOH services 4-5 times, compared with those who reported no anxiety or depression (Table 5). Extensive use of OOH services among people with mental problems and psychiatric disease is well documented in previous research [18,30,35,36].

In many countries, Norway included, there is concern about unnecessary use of emergency medical services [19]. Patients, as well as GPs, consider worry an important reason for contact [37,38]. From a medical perspective, most OOH use is non-urgent, and motives for contacting OOH services are primarily patient-related [21,38]. Self-reported anxiety/ depression and the use of health apps might be such patient-related factors. A common feature of apps, OOH services, and anxiety/depression is that they operate independently of opening hours. We consider this a possible explanation of the association between the use of health apps and the use of OOH services, as well as the association between anxiety/depression and the use of OOH services. Unsatisfactory design and functionality of many apps might also partly explain this finding [39]. Furthermore, a lack of GP availability or accessibility during daytime might contribute in some regions [19,38,40].

The extensive use of OOH services among people with T1D, and particularly among those reporting anxiety/depression, indicates that some patients do visit OOH services rather than waiting for a consultation with the regular physician. This might indicate that policy makers and GPs should consider increasing the regular GPs’ capacity and/or extending opening hours. In this regard, eHealth consultations could be a valuable supplement to face-to face consultations.

Many studies regarding mobile apps and worries, anxiety, or emotional distress focus on apps as possible tools in coping with these problems [41]. However, some have studied the potential of digital devices to create or intensify worries and anxiety in susceptible individuals. Recent research found that the Internet has the potential to reduce as well as exacerbate health anxiety [42-44] and that individuals with moderate to high levels of health anxiety experienced more anxiety during and after online symptom checking, whereas those with low illness anxiety experienced relief [44]. Thus, the use of eHealth might as a side effect intensify health anxiety and, used in the wrong context, cause harm instead of benefit to health. If this is the case, it might contribute to explaining the positive association between the use of health apps and the use of OOH services.

Many apps used for monitoring of disease may meet the definition of a medical device [45]. Still, most apps have not been evaluated by authorization authorities and are not CE-marked (Conformité Européenne, European Conformity) according to European Union directives, or regulated by the US Food and Drug Administration [45,46]. Possible risks related to the app itself have been focused [39]; however, little is known about the use of apps outside tightly controlled research settings [34]. A wider focus on effectiveness related to the use by different individuals in different settings has not been addressed thoroughly [42,43,47]. In our opinion, such possible side effects should be evaluated for all equipment considered as medical devices, apps included.

### Anxiousness and Change of Health Care Seeking Behavior Based on Internet Information

We found that 31.8% (115/487) of people with T1D and 44.5% (57/128) of the OOH users had felt anxious based on information from the Internet (Table 1). According to data collected by the Tromsø Study (general population), 25.8% had felt anxious [27], whereas 38.0% among elderly people in the Netherlands had felt anxious based on information from the Internet [16]. Our figures for people with T1D are placed between them, whereas figures for the OOH users exceeds the other rates.

The differences between the T1D sample and the OOH users among them were greater for feeling anxious (31.8% vs 44.5%) than for feeling reassured (54.6% vs 55.9%), which might indicate that using the Internet for health purposes could have more negative effects for the OOH users, compared with the T1D sample. Even if many people reported anxiety, more people had felt reassured based on Internet information in our study, as well as in the Tromsø Study and in the Dutch study (47.7% and 56.0%, respectively) [16,27].

We found that 17.5% (87/497) of the total T1D sample and 28.7% (37/129) of the OOH users among them decided to consult a doctor based on Internet information—when they would otherwise not have consulted one (Table 1). The similar rate from the Tromsø Study data was 23.6% [27]. Again, our rate for the OOH users exceeds the rates for the total T1D sample, as well as the general population rate obtained from the Tromsø Study. This might indicate that information from the Internet has a larger influence on the OOH users in this regard. Among elderly people in the Netherlands, almost half of the participants (48%) had decided to consult a doctor based on Internet information, which might be explained by cultural diversities, differences in the use and in trusting information from the Internet, as well as methodological differences [16].

Those who had felt anxious based on information from the Internet were 2-3 times more likely to visit OOH services than those who had not felt anxious. People who had decided to consult a doctor based on information from the Internet—when they would otherwise not have consulted one—were almost three times as likely to visit OOH services compared with those who had not changed their decision regarding consulting a doctor. A recent systematic review and meta-analyses found a positive correlation between health anxiety and online health information seeking [48]. As health anxiety levels increase, the relationship between health information seeking online and visiting a doctor based on information found online also increases [42,49]. Other studies asked whether use of the Internet had changed the frequency of doctor visits. Around 90% in these general population studies reported that use of the Internet for health purposes did not change their health care seeking behavior (United States 94%, Japan 88.9%, France 88.6%) [50-52]. Consequently, around 10% did change their health care seeking behavior, as they made either more visits or fewer visits due to information from the Internet. However, other available studies in different populations, most of them general populations, cannot be directly compared with the current study of people with T1D. Our findings may suggest that when decisions to consult a doctor are based on Internet information, people tend to consult as soon as possible. Since OOH services are available at any time, they might be a natural choice.

### Strengths and Limitations

Strengths and limitations have been explored in detail in our first study in this project [14]. The strengths of this study are similar to the strengths discussed in the first paper in this project [14]. The most important strength is the focus on a scarcely investigated research field, which might contribute to evidence-based planning for future health care services in a society where eHealth is increasingly used. Other strengths are the detailed questionnaire specifically tailored to people with diabetes, the recruitment of participants from all of Norway, the inclusion of a wide age span of participants, and that we were able to analyze the data shortly after they were collected. Finally, the collection of data in cooperation with NDA enabled us to develop excellent user participation with a large and important group of health care users.

A limitation of this study is the low estimated participation rate. However, response rate must not be confused with response quality [28]. We found that older people dominated among the late respondents compared with the early respondents [14]. As late respondents might be more similar to non-respondents, younger individuals might be overrepresented.

Distribution of the questionnaire by email is another limitation, which excluded those who do not use the Internet or do not have an email address. Since 97% of Norwegian households have Internet access, we do not think that this affected our results significantly [14]. It is well known that women, healthier persons, higher socioeconomic groups, and middle-aged people are more likely to participate in surveys [14]. This suggests that women, people around 40 to 80 years, people in better health, and higher socioeconomic groups might be overrepresented in our study, thus tending to level out a possible skewness in the opposite directions.

Other relevant limitations were recall bias, the validity of self-reported data, and the cross-sectional study design, as reported and discussed in detail in the first study in the DIAcare project [14]. It is not possible to judge the magnitude or direction of a possible non-response bias, since different factors might pull the tendency in different directions or level each other out. The low response rate is in itself not an indication of low representativeness, as non-response bias may be a problem even if response rates are high [53]. We suggested that non-response bias posed a limited threat to our study’s validity; however, generalization must be made with caution.

Increasing travel distance is associated with reduced use of OOH services in Norway [54]. The lack of information about travel distance is thus a study limitation. However, we have no reasons to believe that the use of apps or other of the independent variables are influenced by travel distance and do not consider this a confounding threat to our results.

It should also be mentioned that worries/anxiety/ depression/emotional distress are not defined according to diagnostic manuals in this study and rely solely on self-report. We consider self-report to be interesting as such in this field and do not think that this has disturbed the validity of our results.

### Future Research

This study investigated the use of health apps in general and associations with the use of OOH services. For future research, the more specific use of diabetes self-care apps and associations with the use of different health care services would be beneficial. Nor did this study investigate the reasons for visiting OOH services, which would be interesting as well: were visits directly related to the use of eHealth for diabetes self-care, or were they due to other health care needs? Furthermore, the finding that information acquired from the Internet is associated with making decisions to attend OOH services merits further investigations.

### Conclusions

We found that people with T1D were extensive users of OOH services and that OOH service users were extensive users of eHealth. There was a positive association between the use of OOH services and the use of health apps, as well as between the use of OOH services and self-reported anxiety or depression. Feeling anxious based on information from the Internet was positively associated with visiting OOH services. Likewise, there was a positive association between deciding to consult a doctor based on information from the Internet and the probability of visiting OOH services. The use of eHealth seems to have a large impact on people with T1D. This study investigated the use of health apps in general, and we think that the more specific use of diabetes self-care apps and associations with the use of different health care services would be of interest for future research. Decision making regarding doctor visits based on information from the Internet also merits further investigations, along with specific investigations regarding reasons for visiting.

## Appendix

Multimedia Appendix 1 Questionnaire.

## Abbreviations

CI: confidence interval
GP: general practitioner
NDA: Norwegian Diabetes Association
NSD: Norwegian Social Science Data Service
REK: Regional Committee for Medical and Health Research Ethics
OOH: out-of-hours
OR: odds ratio
T1D: type 1 diabetes

## References

[1] Ogurtsova K, da Rocha Fernandes JD, Huang Y, Linnenkamp U, Guariguata L, Cho NH, Cavan D, Shaw JE, Makaroff LE (2017). IDF Diabetes Atlas: Global estimates for the prevalence of diabetes for 2015 and 2040. Diabetes Res Clin Pract.

[2] Norwegian Institute of Public Health.

[3] Sandahl K, Nielsen LB, Svensson J, Johannesen J, Pociot F, Mortensen HB, Hougaard P, Broe R, Rasmussen ML, Grauslund J, Peto T, Olsen BS (2017). Increased mortality in a Danish cohort of young people with Type 1 diabetes mellitus followed for 24 years. Diabet Med.

[4] Jenssen TG, Tonstad S, Claudi T, Midthjell K, Cooper J (2008). The gap between guidelines and practice in the treatment of type 2 diabetes A nationwide survey in Norway. Diabetes Res Clin Pract.

[5] Mouland G (2014). [Diabetes in general practice--were treatment goals reached?]. Tidsskr Nor Laegeforen.

[6] Cooper JG, Claudi T, Thordarson HB, Løvaas KF, Carlsen S, Sandberg S, Thue G (2013). Treatment of type 1 diabetes in the specialist health service--data from the Norwegian Diabetes Register for Adults. Tidsskr Nor Laegeforen.

[7] World Health Organization.

[8] Statistics Norway Technology and Innovation.

[9] Statistics Norway Internet and Mobile Phones.

[10] Wangberg SC, Andreassen HK, Prokosch H, Santana SMV, Sørensen T, Chronaki CE (2008). Relations between Internet use, socio-economic status (SES), social support and subjective health. Health Promot Int.

[11] Andreassen HK, Bujnowska-Fedak MM, Chronaki CE, Dumitru RC, Pudule I, Santana S, Voss H, Wynn R (2007). European citizens' use of E-health services: a study of seven countries. BMC Public Health.

[12] Bujnowska-Fedak MM (2015). Trends in the use of the Internet for health purposes in Poland. BMC Public Health.

[13] Fox S, Duggan M (2013). Health Online.

[14] Hansen AH, Broz J, Claudi T, Årsand E (2018). Relations Between the Use of Electronic Health and the Use of General Practitioner and Somatic Specialist Visits in Patients With Type 1 Diabetes: Cross-Sectional Study. J Med Internet Res.

[15] Sørensen T, Andreassen HK, Wangberg SC (2014). Prosjektrapport e-helse i Norge 2013.

[16] Medlock S, Eslami S, Askari M, Sent D, de Rooij SE, Abu-Hanna A (2013). The consequences of seniors seeking health information using the internet and other sources. Stud Health Technol Inform.

[17] Hansen AH, Kristoffersen AE, Lian OS, Halvorsen PA (2014). Continuity of GP care is associated with lower use of complementary and alternative medical providers: a population-based cross-sectional survey. BMC Health Serv Res.

[18] Sandvik H, Hunskaar S (2018). Frequent attenders at primary care out-of-hours services: a registry-based observational study in Norway. BMC Health Serv Res.

[19] Sandvik H, Hunskår S, Diaz E (2012). Use of emergency medical services by patients encompassed by the Regular GP scheme. Tidsskr Nor Laegeforen.

[20] Statistics Norway (2018). General practitoners services.

[21] Thoresen CK, Sandvik H, Hunskaar S (2016). Cancer patients' use of primary care out-of-hours services: a cross-sectional study in Norway. Scand J Prim Health Care.

[22] Catan G, Espanha R, Mendes RV, Toren O, Chinitz D (2015). Health information technology implementation - impacts and policy considerations: a comparison between Israel and Portugal. Isr J Health Policy Res.

[23] Cline RJ, Haynes KM (2001). Consumer health information seeking on the Internet: the state of the art. Health Educ Res.

[24] Hansen AH, Bradway M, Broz J, Claudi T, Henriksen O, Wangberg SC, Årsand Eirik (2016). The Use of eHealth and Provider-Based Health Services by Patients with Diabetes Mellitus: Protocol for a Cross-Sectional Study. JMIR Res Protoc.

[25] (2018). The Norwegian Diabetes Association: Årsberetning 2017.

[26] Medlock S, Eslami S, Askari M, Arts DL, Sent D, de Rooij SE, Abu-Hanna A (2015). Health information-seeking behavior of seniors who use the Internet: a survey. J Med Internet Res.

[27] The Tromsø Study.

[28] Johnson TP, Wislar JS (2012). Response rates and nonresponse errors in surveys. JAMA.

[29] Sandvik H, Hunskår S, Blinkenberg J (2017). Årsstatistikk fra legevakt 2017. Report #2-2018.

[30] den Boer-Wolters D, Knol MJ, Smulders K, de Wit NJ (2010). Frequent attendance of primary care out-of-hours services in the Netherlands: characteristics of patients and presented morbidity. Fam Pract.

[31] Washington R, Andrews R, Mutter R Emergency department visits for adults with diabetes, 2010 statistical brief #167.

[32] Li J, Theng Y, Foo S (2016). Predictors of online health information seeking behavior: Changes between 2002 and 2012. Health Informatics J.

[33] Ernsting C, Dombrowski SU, Oedekoven M, O Sullivan JL, Kanzler M, Kuhlmey A, Gellert P (2017). Using Smartphones and Health Apps to Change and Manage Health Behaviors: A Population-Based Survey. J Med Internet Res.

[34] Trawley S, Baptista S, Browne JL, Pouwer F, Speight J (2017). The Use of Mobile Applications Among Adults with Type 1 and Type 2 Diabetes: Results from the Second MILES-Australia (MILES-2) Study. Diabetes Technol Ther.

[35] Dent A, Hunter G, Webster AP (2010). The impact of frequent attenders on a UK emergency department. Eur J Emerg Med.

[36] Flarup L, Moth G, Christensen MB, Vestergaard M, Olesen F, Vedsted P (2014). Chronic-disease patients and their use of out-of-hours primary health care: a cross-sectional study. BMC Fam Pract.

[37] Keizer E, Maassen I, Smits M, Wensing M, Giesen P (2016). Reducing the use of out-of-hours primary care services: A survey among Dutch general practitioners. Eur J Gen Pract.

[38] Keizer E, Smits M, Peters Y, Huibers L, Giesen P, Wensing M (2015). Contacts with out-of-hours primary care for nonurgent problems: patients' beliefs or deficiencies in healthcare?. BMC Fam Pract.

[39] Brzan PP, Rotman E, Pajnkihar M, Klanjsek P (2016). Mobile Applications for Control and Self Management of Diabetes: A Systematic Review. J Med Syst.

[40] Zhou Y, Abel G, Warren F, Roland M, Campbell J, Lyratzopoulos G (2015). Do difficulties in accessing in-hours primary care predict higher use of out-of-hours GP services? Evidence from an English National Patient Survey. Emerg Med J.

[41] Van Ameringen M, Turna J, Khalesi Z, Pullia K, Patterson B (2017). There is an app for that! The current state of mobile applications (apps) for DSM-5 obsessive-compulsive disorder, posttraumatic stress disorder, anxiety and mood disorders. Depress Anxiety.

[42] Singh K, Brown RJ (2014). Health-related internet habits and health anxiety in university students. Anxiety Stress Coping.

[43] Singh K, Fox J, Brown RJ (2016). Health anxiety and Internet use: A thematic analysis. Cyberpsychology: Journal of Psychosocial Research on Cyberspace.

[44] Doherty-Torstrick ER, Walton KE, Fallon BA (2016). Cyberchondria: Parsing Health Anxiety From Online Behavior. Psychosomatics.

[45] Holubova A, Bradway M, Årsand E, Hallgren D, Hartvigsen G (2015). Do mobile medical apps need to follow European and US regulations or not: decisions exemplified by diabetes management app.

[46] Krieger WH (2016). When are medical apps medical? Off-label use and the Food and Drug Administration. Digit Health.

[47] McKay FH, Cheng C, Wright A, Shill J, Stephens H, Uccellini M (2018). Evaluating mobile phone applications for health behaviour change: A systematic review. J Telemed Telecare.

[48] McMullan M (2006). Patients using the Internet to obtain health information: how this affects the patient-health professional relationship. Patient Educ Couns.

[49] Eastin MS, Guinsler NM (2006). Worried and wired: effects of health anxiety on information-seeking and health care utilization behaviors. Cyberpsychol Behav.

[50] Baker L, Wagner TH, Singer S, Bundorf MK (2003). Use of the Internet and e-mail for health care information: results from a national survey. JAMA.

[51] Takahashi Y, Ohura T, Ishizaki T, Okamoto S, Miki K, Naito M, Akamatsu R, Sugimori H, Yoshiike N, Miyaki K, Shimbo T, Nakayama T (2011). Internet use for health-related information via personal computers and cell phones in Japan: a cross-sectional population-based survey. J Med Internet Res.

[52] Beck F, Richard J, Nguyen-Thanh V, Montagni I, Parizot I, Renahy E (2014). Use of the internet as a health information resource among French young adults: results from a nationally representative survey. J Med Internet Res.

[53] Halbesleben JRB, Whitman MV (2013). Evaluating survey quality in health services research: a decision framework for assessing nonresponse bias. Health Serv Res.

[54] Raknes G, Morken T, Hunskår S (2014). [Travel distance and the utilisation of out-of-hours services]. Tidsskr Nor Laegeforen.

